# Improving and Maintaining Winter Hardiness and Frost Tolerance in Bread Wheat by Genomic Selection

**DOI:** 10.3389/fpls.2019.01195

**Published:** 2019-10-01

**Authors:** Sebastian Michel, Franziska Löschenberger, Jakob Hellinger, Verena Strasser, Christian Ametz, Bernadette Pachler, Ellen Sparry, Hermann Bürstmayr

**Affiliations:** ^1^Department of Agrobiotechnology, IFA-Tulln, University of Natural Resources and Life Sciences Vienna, Tulln, Austria; ^2^Saatzucht Donau GesmbH. & CoKG, Probstdorf, Austria; ^3^C&M Seeds, Palmerston, ON, Canada

**Keywords:** bread wheat, cold tolerance, copy number variation, genomic prediction, winter survival

## Abstract

Winter hardiness is a major constraint for autumn sown crops in temperate regions, and thus an important breeding goal in the development of new winter wheat varieties. Winter hardiness is though influenced by many environmental factors rendering phenotypic selection under field conditions a difficult task due to irregular occurrence or absence of winter damage in field trials. Controlled frost tolerance tests in growth chamber experiments are, on the other hand, even with few genotypes, often costly and laborious, which makes a genomic breeding strategy for early generation selection an attractive alternative. The aims of this study were thus to compare the merit of marker-assisted selection using the major frost tolerance QTL *Fr-A2* with genomic prediction for winter hardiness and frost tolerance, and to assess the potential of combining both measures with a genomic selection index using a high density marker map or a reduced set of pre-selected markers. Cross-validation within two training populations phenotyped for frost tolerance and winter hardiness underpinned the importance of *Fr-A2* for frost tolerance especially when upweighting its effect in genomic prediction models, while a combined genomic selection index increased the prediction accuracy for an independent validation population in comparison to training with winter hardiness data alone. The prediction accuracy could moreover be maintained with pre-selected marker sets, which is highly relevant when employing cost reducing fingerprinting techniques such as targeted genotyping-by-sequencing. Genomic selection showed thus large potential to improve or maintain the performance of winter wheat for these difficult, costly, and laborious to phenotype traits.

## Introduction

Winter hardiness in wheat is a complex trait that is strongly influenced by a multitude of environmental factors such as the presence of a snow cover, soil fertility, soil heaving or ice encasement, as well as biotic factors like disease pressure or insect damage (Fowler et al., 1977). Tolerance to temperatures below freezing is though often of pivotal importance for the survival across winter, aside from the other mentioned indirect effects (Limin and Fowler, 1991). The ability to withstand prolonged exposure to low temperatures can substantially vary between genotypes (Fowler and Gusta, 1979; Skinner and Garland-Campbell, 2008a), and is among others associated with their particular vernalization requirement (Koemel et al., 2004) and a differential reaction to repeated thaw-freeze cycles that might occur in the field (Skinner and Bellinger, 2016). Despite the existing variation for winter hardiness and frost tolerance in wheat (Fowler et al., 1977; Longin et al., 2013; Sthapit Kandel et al., 2018), the improvement for these traits has been rather limited, and efforts to enhance them by using exotic sources with excellent winter hardiness like rye have been less promising (Limin and Fowler, 1991). One major reason for this limited genetic progress can be seen in the difficult phenotyping and lack of information concerning winter hardiness when conducting selection decisions caused by the absence or irregular occurrences of winter damage in field trials (Beil et al., 2019) that are in some years replaced by complete winter kill of an entire plant stand (Fowler, 1979). 

The presence of non-systematic trends like soil heterogeneity and differential snow cover within field trials can moreover result in biased performance estimates of the selection candidates, which requires experimental layouts that allow an appropriate adjustment for these trends, e.g., by applying spatial statistical methods (Burgueño et al., 2000). Alternatives for field trials are given by conducting semi-controlled experiments with snow-out shelters (Hoeser, 1953; Sieber et al., 2014) or controlled frost tolerance experiments in climate chambers with predefined temperature programs (Gusta et al., 1997; Gusta et al., 2001; Skinner and Garland-Campbell, 2008b). The latter methods include the assessment of a lethal temperature at which 50% of the plants die due to freezing (LT_50_) (Gusta et al., 2001), recovery of the plants after prolonged storage at milder freezing temperatures of −5°C (Skinner and Garland-Campbell, 2008b), or exposing plants for a short time span to severe freezing temperature and measuring the percentage of frost damage after a predefined regrowth period (Sutka, 1981). The testing of winter wheat in that manner generally includes a period of cold-hardening (Fowler et al., 1999) for inducing the physiological mechanisms underlying frost tolerance like the accumulation of water-soluble carbohydrates (Fowler et al., 1981; Galiba et al., 1997; Vágújfalvi et al., 1999). Such frost tolerance tests in climate chambers are, however, laborious, costly, and usually limited to few genotypes making a genomic breeding approach an attractive alternative for applied breeding programs.

Hence, numerous studies have been conducted to dissect the genetic architecture of winter hardiness and frost tolerance that revealed the importance of the homologous loci *Vrn-A1*, *Vrn-B1*, and *Vrn-D1* located on the chromosome 5 group both for vernalization response and frost tolerance in wheat (Koemel et al., 2004), with the latter two loci being though fixed for the winter type allele at least in European winter wheat (Langer et al., 2014), although they can be interesting for breeding facultative wheat varieties (Beil et al., 2019). The copy number variation of *Vrn-A1* has, on the other hand, been shown to influence both the vernalization response as well as frost tolerance of winter wheat (Díaz et al., 2012; Zhu et al., 2014; Würschum et al., 2015). Despite lines carrying 1–3 copies of *Vrn-A1*, its copy number variation could though only explain 2.9% of the genetic variance for winter hardiness in a diverse panel of European winter wheat lines (Würschum et al., 2017). Apart from pleiotropic effects of the *Vrn* loci, the *Fr-2B* locus on chromosome 5B has been reported as exclusively being associated with frost tolerance (Tóth et al., 2003). The reduced tolerance to low temperature stress is in this case caused by a loss of function due to the deletion of 11 genes at the locus, which is though very rare in European winter wheat with the frequency of the frost susceptible allele at *Fr-2B* being less than 2% (Pearce et al., 2013). Allele frequencies at the frost tolerance locus *Fr-A2* on chromosome 5A are, on the other hand, more balanced and its larger effect in frost tolerance and winter hardiness both in durum and winter wheat (Sieber et al., 2016; Würschum et al., 2017) makes it a worthwhile target for marker-assisted selection. A map position 30–46 cM proximal to *Vrn-A1* has been determined for *Fr-A2* (Vágújfalvi et al., 2003; Båga et al., 2007), while the locus itself consists of a gene cluster encoding several C-repeat binding factors (CBF) (Vágújfalvi et al., 2005). The copy number variation of these transcriptional activator proteins plays a major role for regulating pathways associated with cold acclimation and frost tolerance (Knox et al., 2010), and specifically the copy number variation of *CBF-A14* showed a strong association with frost tolerance (Soltész et al., 2013) and winter hardiness (Sieber et al., 2016; Würschum et al., 2017). The multi-allelic nature of *CBF-A14* can, however, not be adequately described by bi-allelic SNP markers that are commonly used for fingerprinting in applied plant breeding programs. It has thus been suggested to capture the copy number variation by a haploblock of two SNP markers associated with *Fr-A2*, which explained up to 24% of genetic variance for winter hardiness in bread wheat (Würschum et al., 2017).

Notwithstanding, only a part of the genetic variance can be explained by *Fr-A2* limiting the achievable response to selection in a genomic breeding program, and apart from the few major QTL, many minor QTL have been reported to influence frost tolerance and winter hardiness (Case et al., 2014; Kruse et al., 2017). These loci can be more efficiently targeted by a genomic prediction approach with genome-wide distributed markers (Meuwissen et al., 2001), where a phenotyped and genotyped training population is used to predict the performance of individuals within a validation/selection population by modelling the genetic relationship between both populations (VanRaden, 2008; Piepho, 2009). Utilizing such genomic predictions for selection has been routinely implemented in several national and international wheat breeding programs (Cericola et al., 2017; Jarquin et al., 2017; Belamkar et al., 2018) due to decreasing genotyping costs with new fingerprinting techniques like shallow sequencing (Gorjanc et al., 2017) or with sets of preselected markers in targeted genotyping-by-sequencing (GBS) as well as on custom-made and optimized SNP arrays (Torkamaneh et al., 2018). These advances in genotyping allow thus to conduct an early generation marker-assisted and genomic selection for multiple agronomic traits, which is specifically interesting for difficult, laborious, and costly to phenotype traits like winter hardiness or frost tolerance. The aims of this study were thus 1) to compare the merit of marker-assisted with genomic selection for winter hardiness and frost tolerance, and 2) to assess the potential of combining both traits with a genomic selection index using a high-density marker map or a reduced set of preselected markers.

## Materials and Methods

### Plant Material and Phenotypic Data

The plant material in this study comprised a total of 504 F_4:6_ and F_5:7_ generation or double haploid winter wheat breeding lines (*Triticum aestivum* L.) from a commercial breeding program that were phenotyped for frost tolerance in 2017 and for their winter hardiness in 2012 and 2018. The lines were derived from 311 different crosses and came from three distinct breeding cycles of the breeding program. A subpopulation of 181 of these lines was scored for winter hardiness in two trial locations in Austria 2012 on a 1–9 scale with 1 designating a very good winter survival, i.e., a dense plant stand, while 9 referred to a complete winter kill of the plants in a given field plot in early spring. The same scoring system was used for the winter hardiness assessment of another subpopulation of 110 lines in one Eastern Canadian location 2018. The breeding lines were tested in yield plots at all individual trial locations. The F_5:7_ and doubled haploid lines were replicated twice and randomized together with the unreplicated F_4:6_ lines in a partially replicated row-column design to allow a correction for spatial trends in the experimental fields. The growing season in all trial locations with winter hardiness data was characterized by periods of very low temperatures with and without snow cover (**Supplement Figure S1**). Additional agronomic information for these 110 lines comprised grain yield (dt ha^−1^), protein content (%), flowering date (days), and plant height (cm) that were available both from low temperature stressed conditions in Eastern Canada as well as under non-stressed conditions from multiple trial locations in Central Europe. 

A third subpopulation of 213 lines was finally assessed for their frost tolerance in a climate chamber experiment at the Agricultural Research Institute of the Hungarian Academy of Sciences in Martonvásár (Hungary) following the protocol outlined by Sutka (1981). Briefly, germinated seeds of the 213 lines were planted in wooden boxes (38 × 26 × 11 cm) with a 4:1 mixture of garden soil and sand. The experiment was laid out as randomized complete block design with four replicates, in which the plants were grown for 7 weeks and subsequently hardened for 1 week with day/night temperatures varying between +3°C and −3°C, followed by 4 days of a constant temperature of −4°C. After hardening, the plants were exposed to a freezing temperature of −16°C for 24 h, after which they were allowed to defrost for 2 days at +1°C. The plants were subsequently trimmed to a height of 3 cm to remove necrotic leaves and ease their regrowth. Frost tolerance was finally assessed as percentage of frost damage after a 21 days regrowth period at a day temperature of 17°C and a night temperature of 16°C. 

### Statistical Analysis of Phenotypic Data

The phenotypic data for frost tolerance was analyzed with a linear mixed model of the form:

(1) $${\text{y}}_{\text{ij}}=\mu +{\text{g}}_{\text{i}}+{\text{b}}_{\text{j}}+{\text{r}}_{\text{ij}}$$

where y_ij_ are the phenotypic observations of the frost damage in percent, µ is the grand mean, and b_j is_ the random effect of the jth block, while the residual effect r_ij_ followed a normal distribution with $r∼\text{N}(0,I\sigma_{\text{r}}^{2})$. The effect g_i_ of the *i*th line was firstly modelled as random to estimate the genetic variance $\sigma_{\text{G}}^{2}$ and subsequently fixed to derive the best linear unbiased estimates (BLUEs). The heritability was estimated by ${\text{h}}^{2}=\sigma_{\text{G}}^{2}/(\sigma_{\text{G}}^{2}+\frac{1}{2}\mathrm{MVD})$, where $\sigma_{\text{G}}^{2}$ designates the genetic variance and MVD the mean variance of a difference of the BLUEs (Piepho and Möhring, 2007). 

The phenotypic data for winter hardiness and other agronomic traits from the individual trials 2012 and 2018 were firstly analyzed with various models correcting for row and/or column effects as well as autoregressive variance–covariance structure of the residuals (Burgueño et al., 2000), and the model with the best fit was chosen by Akaike´s Information Criterion (AIC). BLUEs and the heritability were estimated as beforehand, while trials with a heritability larger than 0.3 were considered for further analysis. It should be noticed that a common error variance between the unreplicated tested F_4:6_ breeding lines and replicated F_5:7_ or double haploid breeding lines was assumed in order to estimate heritabilities for each individual trial. Given that a trait was assessed in more than one trial, an across-trial analysis was carried out in the second stage with a linear mixed model of the form:

(2) $${\text{y}}_{\text{ij}}=\mu +{\text{g}}_{\text{i}}+{\text{t}}_{\text{j}}+{\text{e}}_{\text{ij}}$$

where y_ij_ are the BLUEs for the respective trait from the first stage, µ is the grand mean, and g_i_ is the effect of the ith line. The effect of the jth trial t_j_ was fixed, while the effect e_ij_ that incorporated both the trial-by-line interaction variance and the residual effect was assumed random and followed a normal distribution with $r∼\text{N}(0,\text{I}\sigma_{\text{e}}^{2})$. The heritability for the analysis of individual trials and across trials was determined as described for the frost tolerance experiment. All phenotypic analyses were conducted using the statistical package *ASReml* for the R programming environment (R Development CoreTeam, 2018).

### Genotypic Data and Population Structure

Leaf samples from the 504 lines were collected from a minimum of 10 plants and used for DNA extraction with the protocol outlined by Saghai-Maroof et al. (1984), and each line was subsequently genotyped with the DarT GBS approach (Diversity Arrays Technology Pty Ltd). Quality control was applied by removing markers with a minor allele frequency smaller than 0.10 as well as more than 10% of missing data. The pair-wise correlation between markers was used as an *ad hoc* measure of linkage disequilibrium, and one marker from each marker pair that had an r² = 1.0 was dropped at random to remove strongly correlated predictor variables for genomic predictions. Additionally, a Kompetitive Allele Specific PCR (KASP) marker analysis was carried out for the markers S2269949, S1077313, S1862541, and S1298957 that were previously reported to be associated with the copy number variation at the frost tolerance locus *Fr-A2* on chromosome 5A (Sieber et al., 2016; Würschum et al., 2017). The *missForest* algorithm Stekhoven and Bühlmann (2012) was used for a chromosome-wise imputation of missing data points for obtaining a final set of 1,413 SNP markers. Two haploblocks were subsequently generated to specifically target the copy number variation at *Fr-A2* as suggested by Sieber et al. (2016) and Würschum et al. (2017). For this purpose, the markers S2269949 and S1077313 were combined to a haploblock designated as *CNV Fr-A2(S)* (Sieber et al., 2016), while S1862541 and S1298957 were combined to *CNV Fr-A2(W)* (Würschum et al., 2017). Given that both flanking markers carried the allele for frost tolerance or susceptibility, the haploblock allele was coded as homozygous in the marker matrix, elsewise it was coded as heterozygous. The population structure with the corresponding membership of each line to its subpopulation was finally investigated by a principal component analysis (**Supplement Figure S2**).

### Marker-Assisted and Genomic Prediction Models

The merit of genomic prediction for winter hardiness and frost tolerance was firstly assessed in a 100 times replicated resampling scheme with the field data from 2012 as well as separately with the measured frost tolerance in the climate chamber experiment 2017. For this purpose, a total of 130 lines were randomly sampled into a training population and 30 lines in a validation population within each of these datasets. The kinship between these lines was estimated by a genomic relationship matrix, which was computed according to the method described by Endelman and Jannink (2012): 

(3) $$K=WW^{\text{T}}/2\Sigma ({\text{p}}_{\text{k}}-1){\text{p}}_{\text{k}}$$

where **W** is a centered N×M marker matrix of the i lines with W_ik_ = Z_ik_ + 1 – 2p_k_ and p_k_ being the allele frequency at the kth locus. Genomic estimated breeding values (GEBV) were afterwards derived by genomic best linear unbiased prediction (GBLUP) models that included the obtained genomic relationship matrix:

(4) $$y=1_{N}\mu +Z_{\text{G}}u_{\text{G}}+r$$

where **y** is an N×1 vector of BLUEs obtained in the phenotypic analysis, **Z_G_** is a random effect design matrix for additive genetic effects, and **u_G_** is an N×1 vector of additive effects with $u_{\text{g}}∼\text{N}(0,K\sigma_{{\text{u}}_{\text{G}}}^{2})$. The residual effect **r** followed a normal distribution $r∼\text{N}(0,I\sigma_{\text{r}}^{2})$, and µ designated the intercept with **1*_N_*** being an N×1 vector where all elements equal 1. Given the prior knowledge about the large influence of the *Fr-A2* locus both on winter hardiness and frost tolerance, upweighting the effects of the *CNV Fr-A2(S)* and *CNV Fr-A2(W)* haploblocks was tested by modeling them as fixed effects in separate weighted genomic best linear unbiased prediction (WBLUP) models (Bernardo, 2014; Zhao et al., 2014):

(5) $$y=1_{N}\mu +M_{\text{Fr}2}\beta_{\text{Fr}2}+Z_{\text{G}}u_{\text{G}}+r$$

where **y** is again an N×1 vector of BLUEs obtained in the phenotypic analysis and **u_G_** an N×1 vector of additive effects, while **M_Fr2_** contained the coding of the haploblock alleles and β**_Fr2_** designated the effect of either *CNV Fr-A2(S)* or *CNV Fr-A2(W)*.

Aside from testing the merit of genomic prediction and its enhancement by integrating the copy number variation at the *Fr-A2* locus, it was of further interest to investigate the possibility to derive a reduced set of markers that would largely maintain the prediction accuracy of the previous models for designing custom SNP chips or targeted GBS. For this purpose, genome-wide association mapping was conducted within each of the training populations of 130 lines based on a linear mixed model following (Yu et al., 2006):

(6) $$\text{y}=1_{N}\mu +\text{M}\alpha +{\text{Z}}_{\text{G}}{\text{u}}_{\text{G}}+\text{r}$$

where α is the fixed marker effect with the corresponding incidence matrix **M** of +1, -1, and 0 coding for homozygous major, minor, and heterozygous, respectively, while the designations of all the other effects were retained from the previous models. Firstly, the 12 markers with the highest significance levels on each chromosome were determined and subject to a chromosome-wise stepwise regression to identify the best fitting model with 1-6 of these markers. This resulted in a reduced marker set of 21-126 genome-wide distributed markers, which was again reduced by a stepwise regression in a second stage to a final set of 21 markers. Hence, the suggested algorithm mainly aimed to find marker-trait associations that covered the whole genome and putatively influence either winter hardiness or frost tolerance. Alternatively, a reduced set of 21 markers was selected by stepwise regression using the 126 genome-wide most significant markers as the initial set. The marker number was chosen to generally test the feasibility of a marker pre-selection for the prediction of winter hardiness and frost tolerance, and might have to be modified for the prediction of other traits. It has to be mentioned though that their absolute number is restricted to the number of lines in a training population when using a stepwise regression. The 1–21 markers with the largest effect as well as sets of randomly sampled markers were subsequently employed for a marker-assisted prediction of the 30 lines in the validation population by a ridge-regression best linear unbiased prediction (RR-BLUP) model:

(7) $$\text{y}=1_{N}\mu +Z_{\text{M}}u_{\text{M}}+r$$

where the matrix **Z_M_** contained the marker codings of the 1–21 markers, and the random marker effects in the vector **u** **_M_** were assumed to follow a normal distribution $u_{\text{M}}∼\text{N}(0,I\sigma_{{\text{u}}_{\text{M}}}^{2})$ with variance $\sigma_{{\text{u}}_{\text{M}}}^{2}$ and $r∼\text{N}(0,I\sigma_{\text{r}}^{2})$. The models for genomic prediction and genome-wide association mapping were fitted with the mixed model package *sommer* (Covarrubias-Pazaran, 2016), the stepwise regression was conducted with the *caret* package (Kuhn, 2008), and the marker-assisted prediction was implemented with the *rrBLUP* package (Endelman, 2011) for R (R Development Core Team, 2018).

### Independent Validation and Genomic Index Selection

The prediction models for winter hardiness and frost tolerance were afterwards tested for their merit in an independent validation using the winter hardiness data collected in Eastern Canada 2018. The prediction models were fitted either with 130 lines with winter hardiness data 2012 or frost tolerance data from the climate chamber experiment in 2017, while 90 lines evaluated in Eastern Canada 2018 served as the validation population. The lines used for model training and validation were randomly sampled from each dataset and the resampling scheme was repeated 100 times as beforehand. It was lastly of interest in this study to investigate the possibility of combining predictions for winter hardiness and frost tolerance to putatively increase the prediction accuracy. The respective predicted values for marker-assisted and genomic selection were to this end combined in a selection index of the form:

(8) $$\text{Inde}{\text{x}}_{\text{G}{\text{S}}_{\text{i}}}={\text{X}}_{\text{WIN}{\text{T}}_{\text{i}}}{\text{b}}_{\text{WINT}}+{\text{X}}_{\text{FROS}{\text{T}}_{\text{i}}}{\text{b}}_{\text{FROST}}$$

where the index value of the ith line was calculated by using the respective index weights b_WINT_ and b_FROST_ for winter hardiness $\left({\text{X}}_{\text{WIN}{\text{T}}_{\text{i}}}\right)$ and frost tolerance $\left({\text{X}}_{\text{FROS}{\text{T}}_{\text{i}}}\right)$. The necessary index weights were obtained by aiming to achieve desired gains for each of the two traits employing:

(9) $$b=G^{-1}a$$

with **b** being a vector of index weights, **a** the vector of desired gains, and G**^–1^** the inverse of the genomic variance–covariance matrix based on the predicted performance values from single trait predictions of the respective traits:

(10) $$\left(\begin{array}{cc} \sigma_{\text{WINT}}^{2} & \sigma \\ \sigma & \sigma_{\text{FROST}} \end{array}\right)$$

with the variances of the predicted performance values for both traits being on the diagonal and covariance on the off-diagonal. The vector of desired gains was set to $a={\left({\text{a}}_{\text{WINT}}=\sqrt{\sigma_{\mathrm{WINT}}^{2}},{\text{a}}_{\text{FROST}}={\sqrt{\sigma_{\text{FROST}}^{2}}}^{}\right)}^{\text{T}}$, which corresponded to a desired gain of one standard deviation for each trait, respectively. The prediction accuracy for frost tolerance and winter hardiness within the dataset 2012 and 2017 as well as for the independent validation 2018 was assessed by correlating the genomic estimated breeding and index values with observed values divided by the square root of the heritability.

## Results

### Variation and Trait Correlations for Winter Hardiness and Frost Tolerance

**Figure 1:**
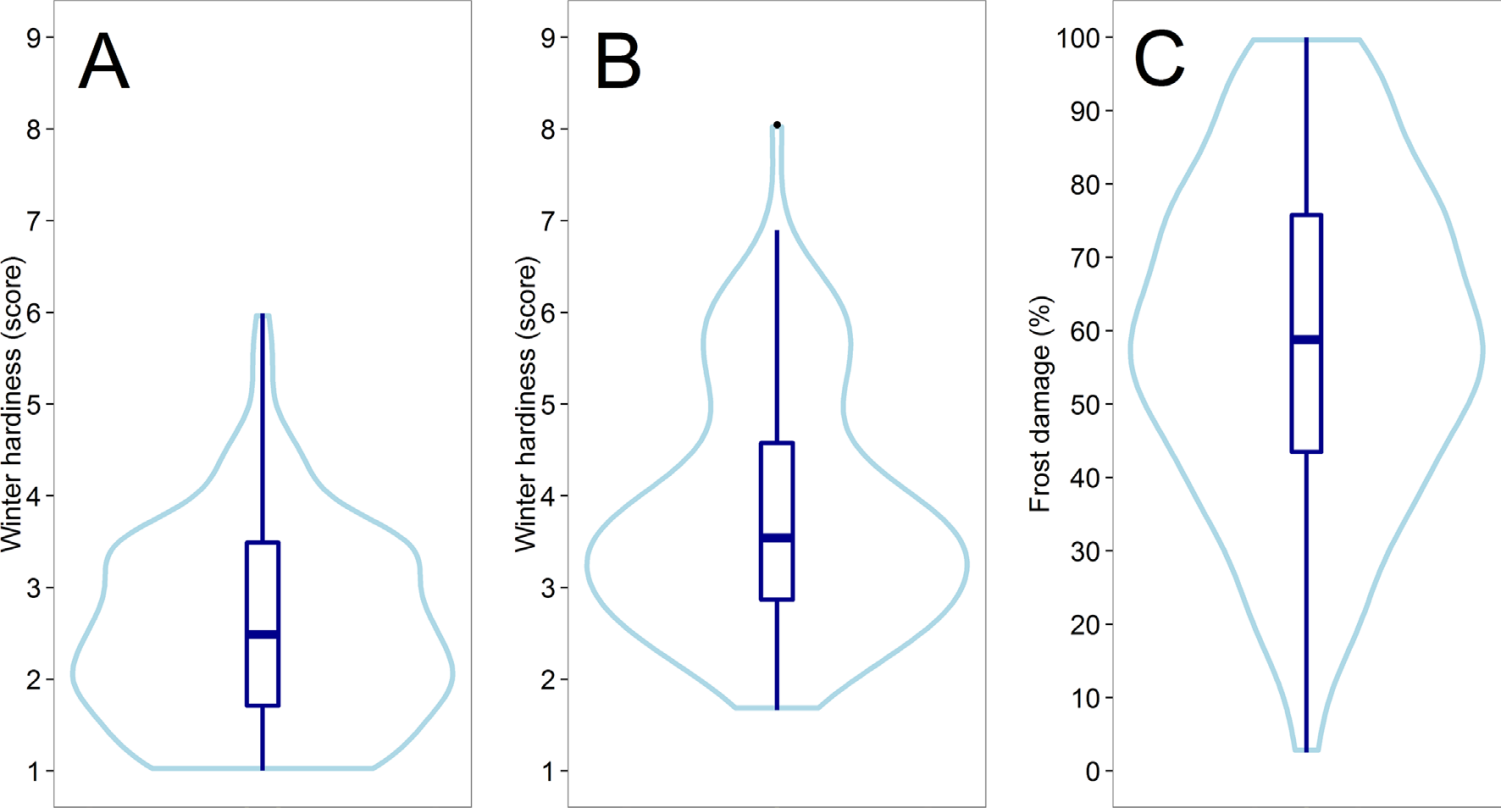
Violin plots showing the distribution of the assessed line performance for winter hardiness in Austria 2012 **(A)** and Eastern Canada 2018 **(B)** as well as for the frost tolerance in the climate chamber experiment 2017 **(C)**.

A medium to high heritability (h² = 0.65 – 0.98) could be achieved for all traits that were assessed in the field trials (**Supplement Table S1**). A large variation could be observed for winter hardiness in the two subpopulations tested in Austria 2012 (h² = 0.71) and Eastern Canada 2018 (h² = 0.65) (**Figures 1A, B**), whereas the subpopulation average in the latter was about 1.3 scoring points higher, though likewise slightly skewed towards increased winter hardiness. The distribution in both environments was furthermore slightly bimodal, which was associated with different haploblock allele at the *Fr-A2* locus (**Supplement Figure S3**). A significant negative correlation was detected between winter hardiness and grain yield in Eastern Canada 2018, i.e., the grain yield of the more winter hardy lines was by trend higher (**Table 1**). Earlier flowering lines appeared furthermore to be more winter hardy under the low temperature stress conditions in Eastern Canada. The relationship with anthesis as well as grain yield became, however, non-significantly different from zero when correlating the winter hardiness of the line with their performance values obtained in the absence of low temperature stress in Central Europe 2018. Artificially induced low temperature stress conditions in the climate chamber experiment 2017 resulted also in high-quality phenotypic data with h² = 0.98 and covered the entire variation for frost tolerance; ranging from hardly any frost damage to complete frost kill (**Figure 1C**). Hence, the phenotypic data had the necessary high quality that was required for further studying the potential of genomic selection for the difficult and costly to assess frost tolerance and winter hardiness.

**Table 1:** Correlation between winter hardiness scoring and other agronomic traits of the 110 lines tested under low temperature stress conditions in Eastern Canada and in the absence of low temperature stress in Central Europe 2018.

	Grain yield	Protein content	Plant height	Anthesis date
Non-stressed conditions	−0.078	−0.322*	−0.270*	−0.055
Stressed conditions	−0.407*	−0.138	−0.348*	0.278*

Winter hardiness was rated on a 1-9 scale (1= very good winter survival, 9 = complete winter kill).

*significant at the 0.01 probability level.

### Marker-Assisted and Genomic Prediction for Winter Hardiness and Frost Tolerance

**Figure 2:**
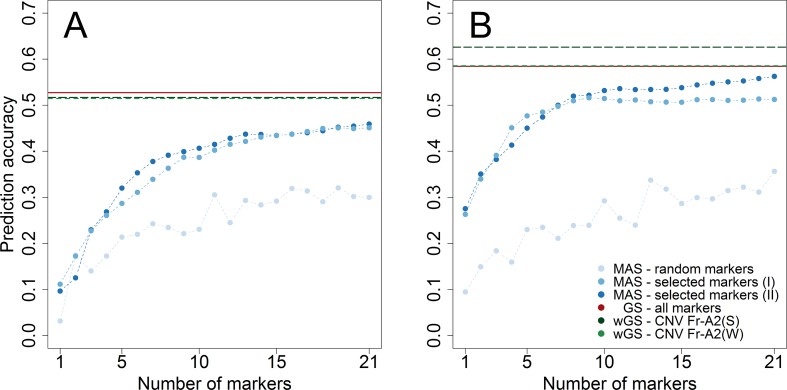
Prediction accuracy assessed by cross-validation within the two subpopulations phenotyped for winter hardiness 2012 **(A)** and frost tolerance 2017 **(B)**. The number of genome wide (I) and chromosome-wise (II) preselected and randomly chosen markers to train prediction models for marker-assisted selection (MAS) varied between 1 and 21, whereas the merit of genomic selection (GS) was assessed by all markers as well in weighted genomic prediction models (wGS) modelling either the haploblock *CNV Fr-A2(S)* (Sieber et al., 2016) or *CNV Fr-A2(W)* (Würschum et al., 2017) as additional fixed effects.

The merit of genomic prediction was firstly assessed by using a resampling scheme within the subpopulations tested for winter hardiness 2012 and frost tolerance 2017. The prediction accuracy for genomic selection was substantially higher than the one for marker-assisted selection with 21 randomly chosen markers in both datasets (**Figure 2**). Notwithstanding, almost the same level of prediction accuracy could be achieved for winter hardiness by marker-assisted prediction with 21 preselected (r_MAS_ = 0.459) as by a genomic prediction that featured more than a thousand markers (r_GS_ = 0.527). A similar observation could be made for the prediction of frost tolerance, where marker-assisted prediction with preselected markers performed merely slightly worse (r_MAS_ = 0.563) than genomic prediction (r_GS_ = 0.585). The latter could be additionally improved by modelling the haploblock *CNV Fr-A2(S)*, associated with the copy number variation at the *Fr-A2* locus, as a fixed effect (r_wGS_ = 0.626). Modelling a haploblock suggested by Sieber et al. (2016) was furthermore beneficial as *CNV Fr-A2(S)* explained a larger proportion of the genetic variance (ρ_G_ = 20.1%) than its component markers S2269949 (ρ_G_ = 16.3%) and S1077313 (ρ_G_ = 13.0%). The haploblock *CNV Fr-A2(S)* suggested by Würschum et al. (2017) did on the other hand not explain a larger proportion of genetic variance (ρ_G_ = 12.6%) than its component marker S1862541 (ρ_G_ = 13.9%) in the study at hand, most likely due to ambiguous allele calls for the other involved marker S1298957 within the investigated breeding germplasm.

**Figure 3:**
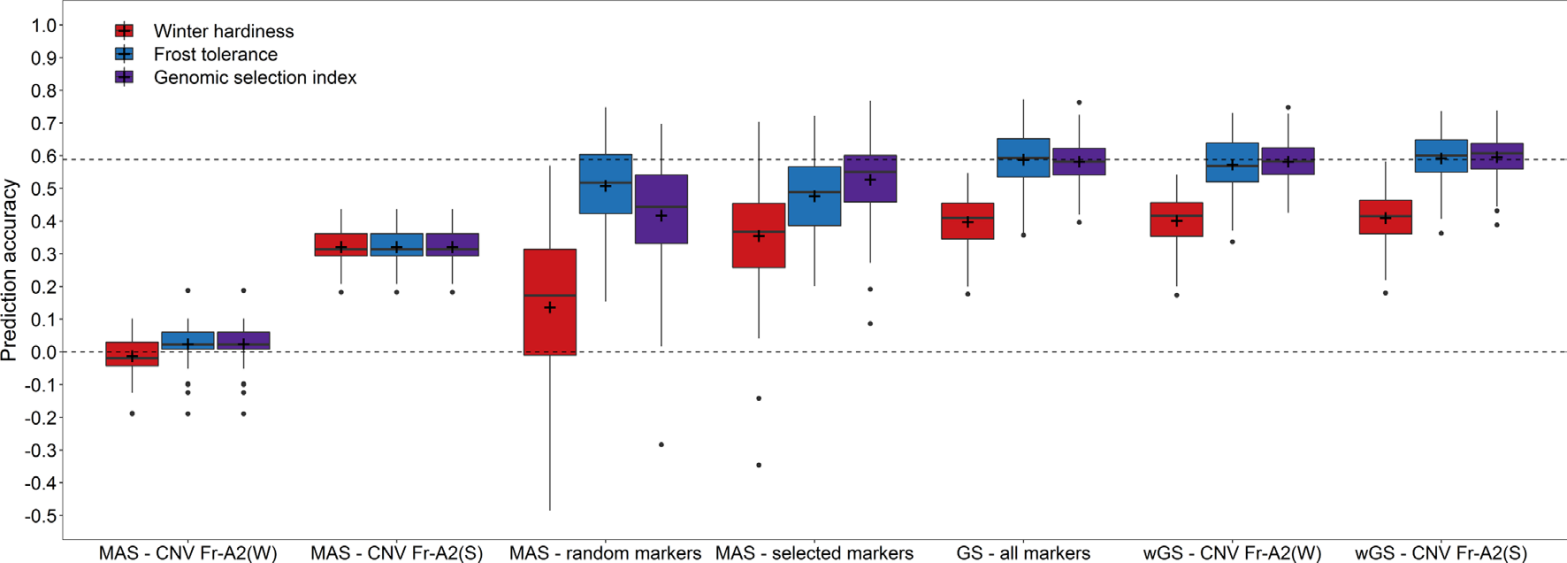
Prediction accuracy for winter hardiness in the independent validation population (2018) using either winter hardiness data (2012) or frost tolerance records (2017) for model training as well as for combining the respective genomic estimated breeding values by a genomic selection index. Prediction models for a marker-assisted selection (MAS) were trained by using only the haploblocks *CNV Fr-A2(S)* (Sieber et al., 2016) and *CNV Fr-A2(W)* (Würschum et al., 2017) or a chromosome-wise preselected set of markers, while the models for genomic selection (GS) were based on all genome-wide distributed markers as well as modelling either the haploblock *CNV Fr-A2(S)* or *CNV Fr-A2(W)* as additional fixed effects in weighted genomic prediction models (wGS).

The effect of the haploblock *CNV Fr-A2(S)* could furthermore be validated in an independent validation using the winter hardiness data collected in Eastern Canada 2018 (**Figure 3**). Upweighting the effect of *CNV Fr-A2(S)* in the prediction models increased the prediction accuracy merely marginally (r_wGS_ = 0.592) in comparison to the basic GBLUP model (r_GS_ = 0.588) when training models with frost tolerance data. The accuracy of predictions based on winter hardiness was generally lower (r_GS_ = 0.398) but could be improved in a WBLUP model (r_wGS_ = 0.410). Combining GEBVs for both traits by a genomic selection index of the respective WBLUP models resulted furthermore beneficial in the highest prediction accuracy among the investigated models (r_GS_ = 0.596). Additionally, the coefficient of variation for the repeated estimates of the prediction accuracy was reduced in the last case, indicating that employing a genomic selection index might lead to more stable and robust performance predictions for winter hardiness. The same pattern could be observed for the marker-assisted prediction with a subset of 21 preselected markers, with which the genomic selection index was nearly as accurate as with the entire set of 1,413 markers available in this study (r_MAS_ = 0.527). It has to be noticed though that the marker subsets mostly differed between the training populations phenotyped either for winter hardiness and frost tolerance so that the actual number of employed markers varied in this scenario between 39 and 42. Nevertheless, the result gave a strong indication that it is feasible to drastically reduce the marker number and at the same time maintain a high prediction accuracy.

## Discussion

The frost tolerance locus *Fr-A2* has been established as a major column in the genetic architecture of winter hardiness and frost tolerance in wheat (Vágújfalvi et al., 2003; Båga et al., 2007; Knox et al., 2010; Zhu et al., 2014; Kruse et al., 2017). Targeting the copy number variation at *Fr-A2* for marker-assisted selection poses, however, a challenge when using SNP-based genotyping platforms. Hence, building haploblocks by combing two markers associated with the expression strength of the putatively causal gene *CBF-14* has been suggested both for durum (Sieber et al., 2016) and winter wheat (Würschum et al., 2017). The explained genetic variance of the suggested haploblock *CNV Fr-A2(S)* was likewise larger than by their component markers in the study at hand, especially for the subpopulation assessed in the climate chamber experiment 2017 (ρ_G_ = 20.3%). The merit of the haploblock *CNV Fr-A2(S)* was though much smaller in explaining the genetic variance for winter hardiness in the field trials from 2012 (ρ_G_ = 3.0%), but again larger in the independent validation population tested in Eastern Canada 2018 (ρ_G_ = 9.2%), which suggests a complex interaction of multiple factors determining winter survival in these trials aside from frost tolerance. Genomic prediction with genome-wide distributed markers resulted thus in higher prediction accuracies than marker-assisted prediction with few significant markers as reported beforehand (Zhao et al., 2013; Würschum et al., 2017). Additionally upweighting the effect of certain markers in genomic prediction models can be worthwhile if their linkage to large effect QTL is known (Rice and Lipka, 2019), which has, e.g., been demonstrated for the *Fr-R2* locus associated with winter survival in rye (Erath et al., 2017) and could be verified for *Fr-A2* in winter wheat in the study at hand (**Figure 2**).

Nevertheless, the advantage of integrating the haploblock *CNV Fr-A2(S)* as fixed effects into the prediction models was rather small in the independent validation, while the prediction accuracy was generally lower for this scenario (**Figure 3**). Independently testing the reliability of genomic predictions is thus advisable especially in practical applications for a complex trait like winter hardiness. A decrease in accuracy is a common problem when genomically predicting across subpopulations tested in different years and is caused among others by genotype–environment interaction as well as an increase in genetic distance between training and validation/selection population (Michel et al., 2016; Schrag et al., 2019). A lower accuracy was thus achieved in the independent validation when training prediction models with the genetically more distant subpopulation assessed for winter hardiness 2012 in comparison to the genetically closer training population phenotyped for frost tolerance 2017 (**Supplement Figure S2**). Several genomic breeding strategies can accordingly be envisaged to select for winter hardy breeding lines. The primary alternative would be to regard the winter hardiness complex as the target trait and directly train prediction models with phenotypic data collected in field trials, which would take into account abiotic factors like freezing temperatures or the presence/absence of a snow cover as well as biotic factors like snow mold diseases (Kruse et al., 2017) that might though vary in their respective importance in different trials and years (Fowler et al., 1977). Updating prediction models can furthermore be difficult in this scenario due to the previously mentioned problems like the irregular occurrence of winter damage in the field, while testing by subcontractors in specific locations known for a more regular occurrence of low temperature stress might be costly but does not guarantee successful experiments. The second alternative would be the assessment of frost tolerance in climate chamber experiments, which can though be laborious and might be costly, especially when initially phenotyping a larger panel of breeding lines to set up a training population. Such frost tests are though reliable in delivering high-quality phenotypic data once a protocol is established, and can be employed for a regular prediction model update with a convenient number of “key individuals” like important crossing parents. A third combined strategy could lastly be envisaged if phenotypic records from both the field and climate chamber are available in a breeding program as in this study. Given the correlation between winter hardiness assessed in field trials and frost tolerance in controlled experiments (Gusta et al., 2001; Erath et al., 2017), combining these traits by a selection index can cover their separate as well as common aspects and enables thus a selection on the total net merit. 

The benefit of this method was particularly apparent in the marker-assisted prediction with preselected markers (**Figure 3**). It has to be noticed though that marker-assisted prediction with few significant markers can suffer from low prediction accuracy, genetic hitchhiking, and quick fixation of favorable alleles. Larger sets of about 20 markers can, on the other hand, already distinguish more than 1 million different combinations, assuming loci are independent, and sampling them systematically from the entire wheat genome resulted in a similar accuracy as for genomic prediction. The chromosome-wise sampling of the most significant markers and selecting among them in a second step was specifically tailored for this study, as it has been shown that frost tolerance in wheat is influenced by loci on at least 10 different chromosomes (Veisz and Sutka, 1998). Notwithstanding, it can be speculated that the implementation of targeted GBS techniques and the development of custom SNP arrays might generally take advantage of such marker selection methods (Cericola et al., 2017; Abed et al., 2018; e Sousa et al., 2019), where several traits have to be considered simultaneously resulting usually in final markers sets of a few hundred instead of thousands of markers. The risk of hitchhiking and allele fixation is accordingly reduced in such a genomic breeding strategy, while the optimal marker set has to be most likely customized for every breeding program. 

Considering multiple traits simultaneously can thus be advantageous for developing an optimal marker set, but it is certainly necessary for identifying breeding lines with a desired combination of traits, which is often challenging, e.g., in the presence of negative trade-offs between major agronomic traits like grain yield and protein content (Rapp et al., 2018; Thorwarth et al., 2019). Regarding winter hardiness and grain yield in this context; it can be expected that more winter hardy cultivars will also have a higher grain yield at the end of the season if low temperature stress conditions have occurred during the winter (Bergjord Olsen et al., 2018) as observed within the subpopulation of breeding lines tested in Eastern Canada 2018. However, it has been stated that winter hardy and frost-tolerant genotypes possess a lower yield potential in years without freezing conditions (Sãulescu and Braun, 2001). The winter 2017/2018 in Eastern Europe was characterized by the absence of such prolonged periods of low temperature stress; however, no significant correlation could be observed between the assessed grain yield in Eastern Europe 2018 and winter hardiness in Eastern Canada 2018 (**Table 1**). Caution must be though taken in the interpretation of this result as severe drought stress afflicted Europe in the cropping season 2017/2018, and the correlation was based on a relative limited number of 130 breeding lines from the same breeding program. Nevertheless, when assembling a broader population with adaptation to different environments ,a negative correlation between winter hardiness and grain yield can be assumed as indicated in a diverse panel of European winter wheat lines that was assessed both for yield components and winter hardiness (Würschum et al., 2017; Würschum et al., 2018). Lines that originated from France and the United Kingdom had a much higher spike fertility than lines from Austria, Poland, and the Czech Republic (Würschum et al., 2018). The lines with the latter origins have though been reported to possess a higher winter hardiness than the lines from the mentioned North Western European countries (Würschum et al., 2017) as, e.g., lines from the United Kingdom are generally higher yielding though less winter hardy as low temperatures are not a major restriction in many growing areas of Great Britain. Hence, the relationship between winter hardiness and grain yield is most likely dependent on the investigated set of lines and their adaptation with regard to winter hardiness and photoperiod sensitivity to specific environments (Gorash et al., 2017). A trade-off between grain yield and frost tolerance was also regularly seen during routine applications of genomic selection in the breeding program from which the here presented data were derived. This trade-off expressed itself as a frequently observed unfavorable correlation between GEBVs for grain yield and frost tolerance, especially when the entire training population employed for the prediction of grain yield was genetically much broader than the populations used in the study at hand. Caution has accordingly to be taken when conducting genomic selection for winter hardiness and frost tolerance, and the usage of genomic selection indices with restrictions or adjusting GEBVs of winter hardiness for grain yield by the residual method (Hänsel, 2001) are possible tools for identifying breeding lines that possess good winter hardiness relative to their yield potential.

## Conclusions

The focus of this study laid on genomic breeding for winter hardiness and frost tolerance in bread wheat. Genomic prediction showed large potential for the selection of these difficult, costly, and laborious to phenotype traits especially when upweighting the effect of the copy number variation at the *Fr-A2* locus and combining predictions in a genomic selection index. The prediction accuracy could moreover be maintained with reduced sets of preselected markers, which is of high relevance when employing cost-reducing fingerprinting techniques such as targeted GBS. A genomic selection of either the best performing lines or a negative selection against the least winter hardy can thus be routinely conducted every year, whereas the absence or irregular occurrence of winter damage in field trials impedes an efficient phenotypic selection for winter hardiness. The large advantage of implementing genomic selection is therefore the availability of information about line performance when conducting selection decision in early generations, which can be used either to maintain or improve this agronomic trait depending on the importance of winter hardiness in a given breeding program.

## Author's note

Genomic predictions for winter hardiness and frost tolerance allow a yearly selection in 14 early generations for these difficult, laborious and costly to phenotype traits.

## Data Availability Statement

The datasets for this manuscript are not publicly available due to commercial interests of the wheat breeding program of Saatzucht Donau GesmbH. & CoKG in which they were generated.

## Ethics Statement

The authors declare that the experiments comply with the current laws of Austria.

## Author Contributions

SM wrote the manuscript and analyzed the data. JH and CA supported in the statistical analysis. JH and VS conducted the Fr-A2 marker analysis. FL, BP, and ES designed the field trials and collected the phenotypic data in the field. FL and HB initiated and guided through the study. All authors read and approved the final manuscript.

## Funding

This research was funded by the “Beyond Europe” FFG project Canadian Hard Red Winter Wheat breeding by genomic tools, lab and field experiments.

## Conflict of Interest

FL, CA, and BP were employed by the company Saatzucht Donau GesmbH. & CoKG. ES was employed by C&M Seeds.

The remaining authors declare that the research was conducted in the absence of any commercial or financial relationships that could be construed as a potential conflict of interest.
