# Supplementary material for: Improving and Maintaining Winter Hardiness and Frost Tolerance in Bread Wheat by Genomic Selection
Source: Front Plant Sci. 2019 Oct 1;10:1195. doi: 10.3389/fpls.2019.01195 (PMC6781858; doi:10.3389/fpls.2019.01195)
Supplement: Supplementary file 1 [file DataSheet_1.pdf]

Supplementary material

Article Title: Improving and maintaining winter hardiness and frost tolerance in bread wheat by genomic selection

Journal: Frontiers in Plant Science – Plant Breeding

Authors: Sebastian Michel, Franziska Löschenberger, Jakob Hellinger, Verena Strasser, Christian Ametz, Bernadette Pachler, Ellen Sparry, Hermann Bürstmayr

Name, affiliation, and email of corresponding author:

Sebastian Michel
Department for Agrobiotechnology (IFA-Tulln)
Institute for Biotechnology in Plant Production
University of Natural Resources and Life Sciences, Vienna (BOKU)
Konrad-Lorenz-Str. 20, 3430 Tulln, Austria
e-mail: sebastian.michel@boku.ac.at

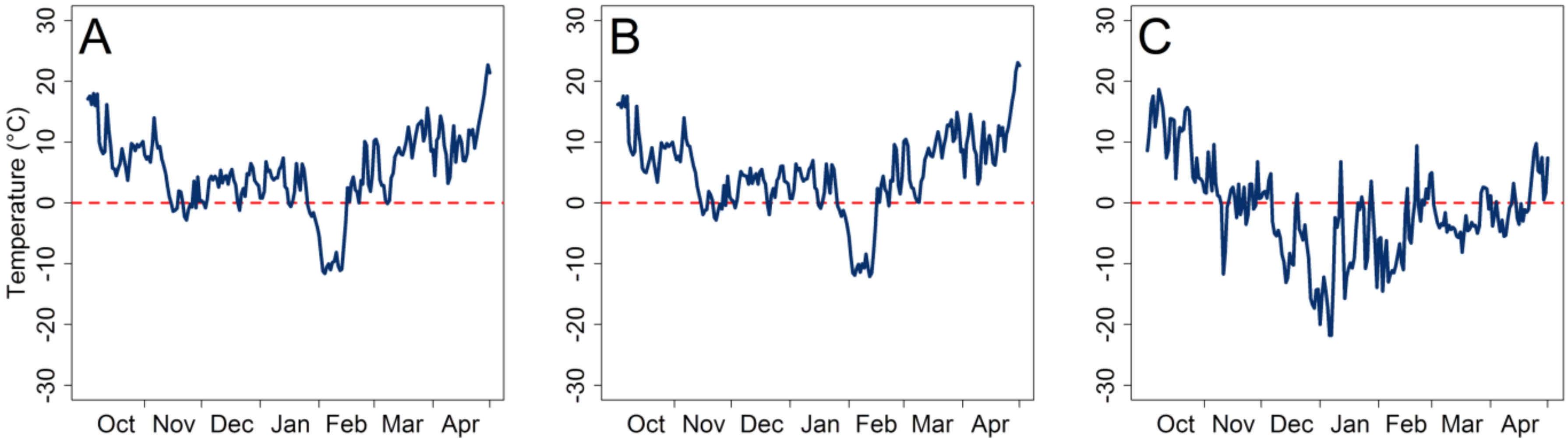

Fig. S1 Average daily temperature at the trial locations Probstdorf (A) and Leopoldsforf (B) in Austria 2012 as well as in Palmerston (Ontario) in Eastern Canada 2018 (C).

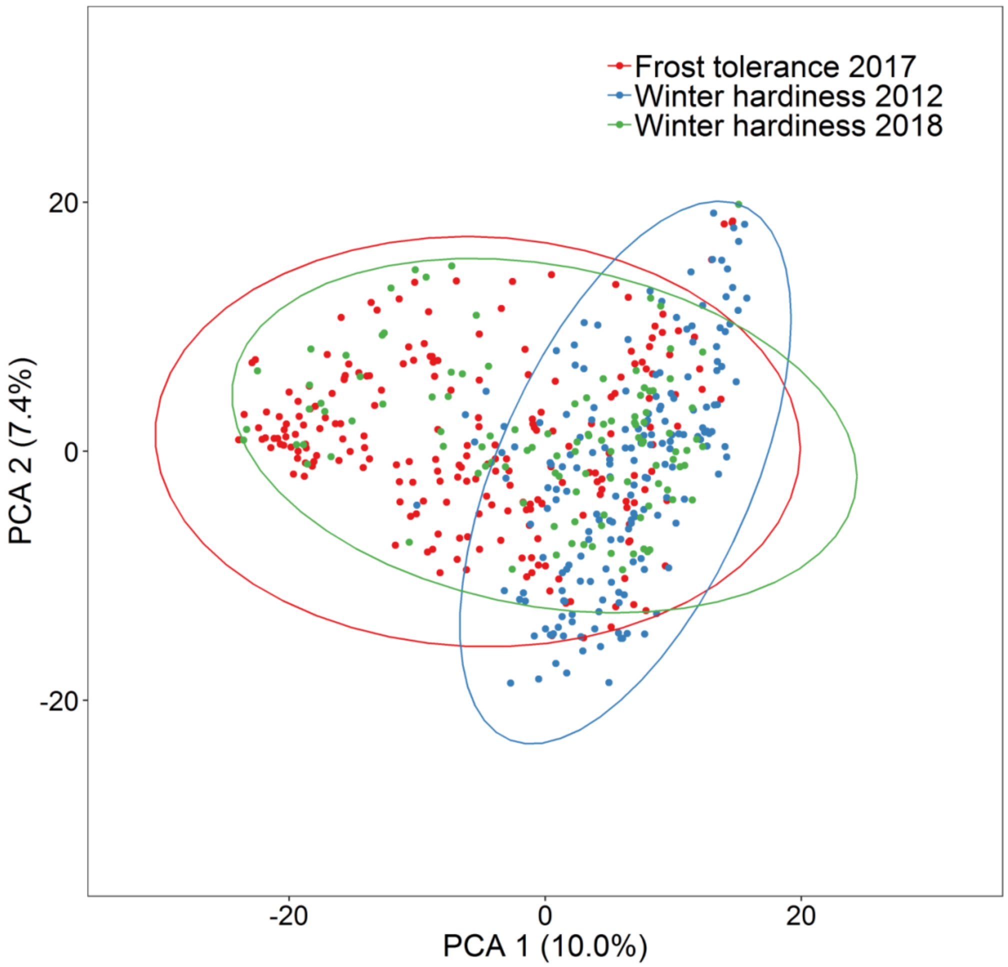

Fig. S2 Population structure of the subpopulations of 181 and 110 scored for winter hardiness in Austria 2012 (Winter hardiness 2012) and Eastern Canada 2018 (Winter hardiness 2018) respectively as well as the subpopulation of 213 lines assessed for their frost tolerance in the climate chamber experiment 2017 (Frost tolerance 2017).

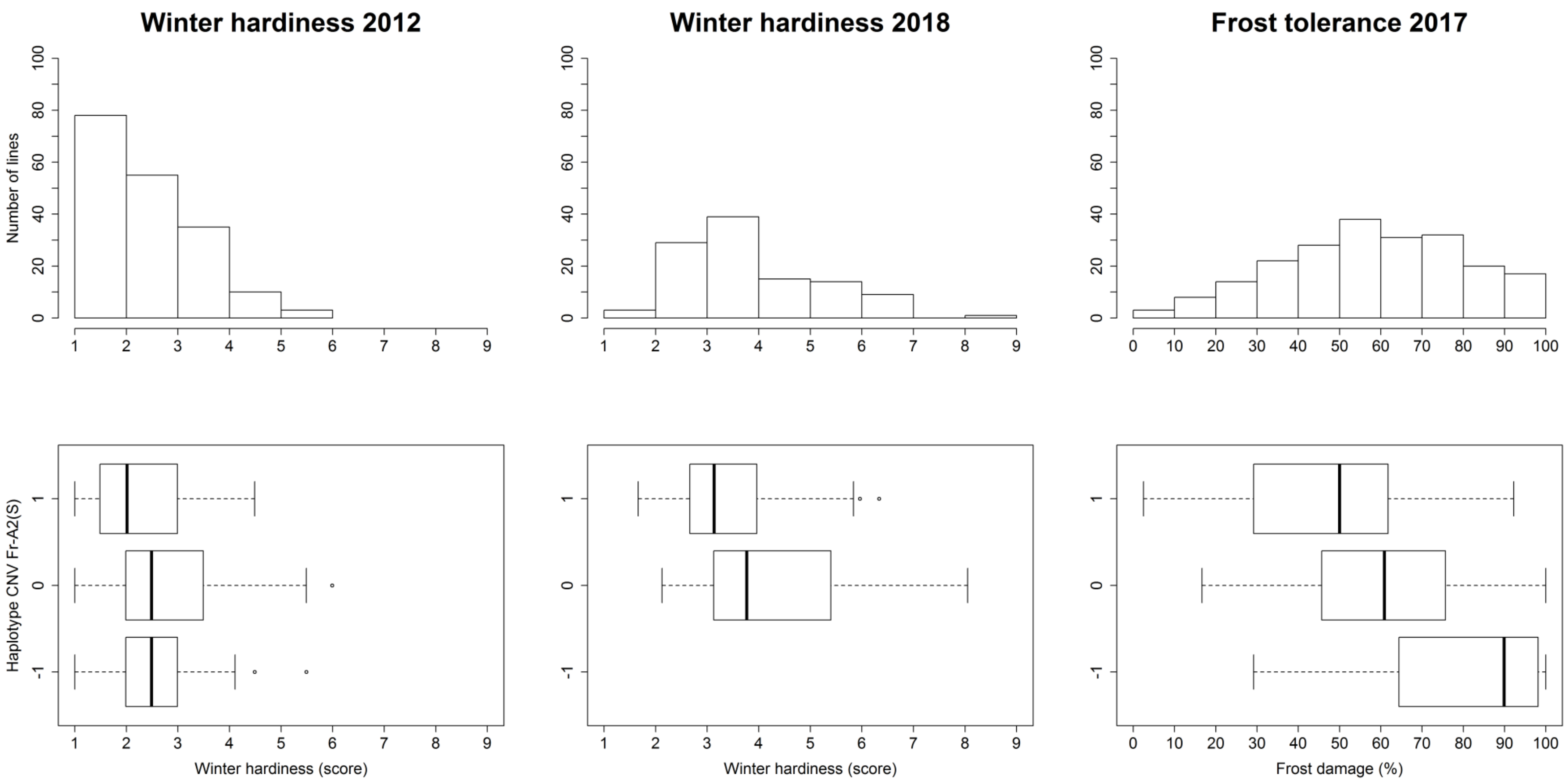

Fig. S3 Relationship between the phenotypic distribution and the haploblock alleles at the *Fr-A2* locus using the markers reported by Sieber et al. (2016) (*CNV Fr-A2(S)*) in the subpopulations of 181 and 110 scored for winter hardiness in Austria 2012 (Winter hardiness 2012) and Eastern Canada 2018 (Winter hardiness 2018) respectively as well as the subpopulation of 213 lines assessed for their frost tolerance in the climate chamber experiment 2017 (Frost tolerance 2017).

Table S1 Mean, range, variance components and heritability for winter hardiness, frost tolerance and other major agronomic traits assessed in the field trials as well as in the climate chamber experiment.

Environment	Trait	Trials (no.)	σ_G^2	σ_e^2	h^2	Min	Mean	Max
Eastern Austria 2012[‡]	Winter hardiness (score)	2	0.76	0.59	0.71	0.9	2.5	6.0
Hungary 2017[†]	Frost damage (%)	1	479.74	30.87	0.98	2.5	58.9	100.0
Eastern Canada 2018[‡]	Winter hardiness (score)	1	1.36	0.64	0.65	1.7	3.8	8.0
	Grain yield (dt ha^{-1})	1	15.87	43.81	0.69	41.5	50.1	64.0
	Protein content (%)	1	0.50	0.18	0.75	11.8	13.9	15.9
	Plant height (cm)	1	35.51	28.99	0.85	60.3	79.5	95.6
	Anthesis date (days)	1	2.98	0.33	0.94	157	161	165
Central Europe 2018[§]	Grain yield (dt ha^{-1})	4	17.27	35.11	0.66	55.3	75.1	84.4
	Protein content (%)	1	0.96	0.48	0.90	12.1	14.3	17.1
	Plant height (cm)	2	22.89	14.98	0.75	90.8	104.2	115.4
	Anthesis date (days)	2	3.81	1.10	0.87	122	126	133

Genotypic variance (σ_G^2), residual variance (σ_e^2), heritability (h^2).
[†] Growth chamber experiment with artificially induced low temperature stress
[‡] Low temperature stress under field trial conditions
[§] Absence of low temperature stress
